# Identifying features of the lentiviral genome crucial for the establishment of latency

**DOI:** 10.1186/1742-4690-10-S1-P107

**Published:** 2013-09-19

**Authors:** Serena Ziglio, Giada Mattiuzzo, Jeremy Luban, Massimo Pizzato

**Affiliations:** 1 Centre of Integrative Biology, University of Trento, Trento, Italy; 2 Division of Virology, National Institute for Biological Standards and Control, MHRA, South Mimms, Potters Bar, Hertfordshire, UK; 3 Program in Molecular Medicine, University of Massachusetts Medical School, Worcester, MA, USA

## Background

Latently infected cells represent a major obstacle to the cure of infection with human immunodeficiency virus type 1 (HIV-1). We have observed that the propensity to establish latency varies greatly among primate lentiviruses. The purpose of our study is to identify the features that determine such variation in order to elucidate the fine molecular mechanisms regulating latency.

## Materials and methods

Using GFP–encoding reporter viruses, the level of infection by SIV, HIV-1 and HIV-2 was quantitated by flow cytometry in cells stably expressing HIV-1 Tat, SIV Tat or vector alone.

## Results

We have observed that SIVmac 239 infects human lymphoid cells much less efficiently than HIV-1. However, ectopic expression of HIV-1 Tat rescued SIV infection.

As host cell type can influence viral gene expression, we tested different cell lines for their ability to support lentiviral latency. In both T (Jurkat TAg and C8166) and T-B hybrid cell lines (CEMX174) SIV exhibited a greater ability to remain transcriptionally silent within the human genome than HIV-1 or HIV-2. The different behaviour in terms of latency was particularly evident in CEMX174 cells, where HIV Tat activation caused more than 20-fold increase in the number of GFP-positive cells infected with SIV, while it had little effect on cells challenged with HIV-1 or HIV-2. The lower level of productive infection displayed by SIVmac239 was not due to a reduced ability of SIV Tat to trigger viral expression in human cells because SIV Tat overexpression reactivated latent SIV as well as HIV Tat did. Moreover, HIV-1 chimeric viruses harboring the U3 region of SIV behaved like the parental HIV-1 viruses, suggesting that viral determinants of SIV latency reside in a part of the lentiviral genome different from the promoter region.

## Conclusions

In human cell lines transduction with SIVmac239 largely establishes a latent infection which is reactivated by overexpressing HIV-1 or SIVmac239 Tat. The tendency of SIVmac239 to establish latent infection is attributable neither to a defective SIV Tat activity nor to a reduced promoter activity of the U3 region in human lymphoid cells. The CEMX174 cell line offers a good model to explore the specific viral determinants which allow SIV to enter latency.

